# Emotionally induced galactorrhoea in a non-lactating female –“Pseudo- Lactation”?

**DOI:** 10.1186/1472-6823-14-98

**Published:** 2014-12-17

**Authors:** Harsha Dissanayake, Sisil Keerthisena, Chinthana Dematapitiya, Prasad Katulanda

**Affiliations:** Diabetes Research Unit, Faculty of Medicine, University of Colombo, Colombo, 7 007 Western Province Sri Lanka; Diabetes Research Unit, Department of Clinical Medicine, Faculty of Medicine, University of Colombo, Colombo, 7 007 Western province Sri Lanka

**Keywords:** Pseudo-lactation, Galactorrhoea, Hyperprolactinaemia

## Abstract

**Background:**

Galactorrhoea is a common clinical problem in endocrinology. Visual and auditory cues from the newborn are known to stimulate prolactin secretion in lactating women. However, hyperprolactinaemia and galactorrhoea in a non-lactating female due to visual and auditory stimuli from an unrelated newborn has not been reported in the past. We report the first such case of ‘pseudo-lactation’.

**Case presentation:**

An 18-year-old girl with type 1 diabetes mellitus presented with galactorrhoea. Apparently galactorrhoea was preceded by seeing the baby, hearing her cries or when remembering her memories. Her menstrual cycles were normal and did not complain of any headache or visual disturbances. She was only on metformin and insulin. Symptoms have rapidly resolved after the newborn was shifted to another location. Examination revealed scanty nipple discharge with gentle pressure. Investigations revealed an elevated serum prolactin of 62.5 ng/mL (2717.4 pmol/L) and fasting plasma glucose of 142 mg/dL (7.9 mmol/L) and HbA1c of 7.6%. Her thyroid function was normal and MRI at the time of galactorrhoea was not available. At 3 months prolactin was normal and MRI revealed only a slight asymmetry of the pituitary without evidence of microadenoma.

**Conclusion:**

The strong temporal relationship between her symptoms and emotional attachment to the newborn with exclusion of other causes on clinical, biochemical and radiological evidence, raise the possibility that transient hyperprolactinaemia was due to a transient lactotroph hyperplasia and hyper function which had been triggered by the stimulatory cues from the newborn.

Emotionally induced “pseudo lactation” may be a rare but important cause for transient hyperprolactinaemia in a non-lactating female.

## Background

Galactorrhoea is defined as milk discharge from nipple in a non-pregnant non-lactating woman. However this is also known to occur in males as well [1]. Galactorrhoea is commoner among females aged 20 – 35 years particularly in previously parous women [1].

Galactorrhoea is often secondary to hyperprolactinaemia [2]. Prolactin hormone secreted by anterior pituitary lactotrophs acts on breast epithelial cells to stimulate milk synthesis. Secretion of prolactin is kept under inhibition by dopamine released from hypothalamus while thyrotrophin releasing hormone (TRH) is known to stimulate prolactin secretion [2]. Prolactin levels in humans show a diurnal pattern; highest levels being observed at night and lowest in the morning [3]. This appears to be independent of sleep pattern and therefore is thought to be true circadian rhythm mediated through the suprachiasmatic nuclei [3].

Effect of emotions and visual, auditory and olfactory stimuli on prolactin secretion are less well documented and are limited, often to animal studies. Happy, romantic or sad emotions have shown to increase serum prolactin levels in women but only marginally and probably to a functionally insignificant level [4]. Stressful life situations have shown mixed results. While some studies have shown increase in prolactin levels [5] others have detected a decline [6]. Academic stress has shown to increase prolactin release in several studies [7]. Olfactory stimuli from pups have shown to increase prolactin levels in lactating rats while ultrasonic auditory stimuli from pups have shown to stimulate prolactin release in both lactating as well as virgin rats [8]. Certain cues from the infant are thought to produce a temporary rise in serum prolactin levels [9]. For example, listening to a recorded infant cry has shown to increase prolactin levels in pregnant and lactating mothers [10]. Similarly, a rise in serum prolactin has been shown in lactating mothers when exposed to their infants [11]. However hormonal responses of non- pregnant and non- lactating women on exposure to infants either in short or long term have not been studied to the best of our knowledge.

Changes in neuroendocrine regulatory systems associated with psychiatric morbidity are also well known. For example major depressive disorders are associated with low dopamine synthesis in the brain [12]. In fact this may at least partly explain the high serum prolactin levels seen in patients with pseudocyesis [13], a rare disorder, commonly associated with major depression, in which the woman thinks she is pregnant without having a foetus in utero, i.e. a ‘pseudo-pregnancy’. However no such equivalent entity is described in medical literature where a woman falsely thinks she is lactating and experience milk production and emotional bonding to an unrelated infant, i.e. a ‘pseudo-lactation’.

We describe the first patient with such symptoms, to the best of our knowledge.

## Case presentation

An 18-year-old girl presented to us complaining of painless bilateral milky nipple discharge for one month. Her menstrual cycles were regular and did not complain of headache or visual disturbance. She was not on any medication except for metformin and insulin for her type 1 diabetes mellitus.

Detailed inquiry revealed that the milky secretion was produced, particularly on seeing a newborn in her next door. The patient sat for long hours in front of a window through which she could always see the infant in the adjoining house. She further described spurting of milk on hearing newborn’s cry or even on remembering its memories. She also claims to dream of herself handling the newborn. She has never experienced similar symptoms before. However her symptoms have rapidly regressed after the neighbours had shifted with the newborn. She is a sexually inactive, school girl preparing for advanced level examination scheduled in 6 months. However she denied any symptoms suggestive of depression, anxiety or delusional disorders.

On examination, she was overweight with a BMI of 25.7 kgm^-2^, well oriented and fluent in speech. She did not have papilloedema, or other focal neurological signs. Her visual fields were normal. Both breasts were in Tanner’s stage 5. By the time of presentation, overt milk discharge had subsided and only a scanty milky nipple discharge was produced with gentle pressure bilaterally.

Investigations revealed an elevated serum prolactin of 62.5 ng/mL (2717.4 pmol/L). Macro-prolactin levels were not measured. Her thyroid function tests and blood biochemistry were normal except for elevated fasting plasma glucose (142 mg/dL/7.9 mmol/L) and HbA1c (7.6%). Unfortunately, MRI scan of the brain was not available during the symptomatic period. Three months after resolution of her symptoms, prolactin levels returned to normal (6.12 ng/mL/266.1 pmol/L). MRI of brain at this point showed only a slight asymmetry of the pituitary gland with a bulky right lobe, without definite evidence of a microadenoma.

## Discussion

Causes for hyperprolactinaemia are numerous; nipple stimulation, chest wall injury, breast feeding, co-morbid psychiatric disorders and treatment with dopamine antagonists are some of them. In addition direct stimulation of lactotrophs by oestrogen (during pregnancy, oestrogen containing contraceptive pills) also causes hyperprolactinaemia [1, 2]. In our patient all above causes except pregnancy have been excluded by a comprehensive history. Pregnancy was excluded by normal ultra sound scan of the abdomen.

Commonest pathological cause for galactorrhoea is a pituitary tumour [14]. Prolactin secreting adenomas or other functional tumours co-secreting prolactin or any form of macroadenoma that is large enough to cause pituitary stalk compression (i.e. disconnection hyperprolactinaemia) may result in hyperprolactinaemia. In this patient MRI scan of the pituitary gland did not reveal any evidence of micro or macroadenoma. Although our patient underwent the MRI scan 4 months after the initial presentation it is highly unlikely for a pituitary tumour to undergo spontaneous regression even without any drugs such as dopamine receptor agonists.

Hypothyroidism removes the negative feedback on hypothalamic TRH and subsequently may cause an increase in prolactin secretion [1, 2]. Nearly 30% of patients with chronic kidney disease have high serum prolactin levels probably secondary to impaired renal excretion of prolactin [15]. In our patient hypothyroidism was excluded by normal range of TSH and T_4_. Although our patient has been diagnosed to have type 1 diabetes mellitus her renal function tests were normal including urine albumin creatinine ratio, serum creatinine and estimated glomerular filtration rate.

Stress is known to cause hyperprolactinaemia. In our patient venipuncture and psychological stressors are important to consider. When withdrawing venous blood for measurement of prolactin levels our patient was subjected to non-stress venipuncture and had only a single prolactin value. Endocrine society clinical practice guidelines also recommend single measurement of serum prolactin without excessive stress for diagnosis of hyperprolactinaemia [16].

Our patient is a schoolgirl preparing for the advanced level examination scheduled in 6 months. Therefore exam stress could have played a major component in her hyperprolactinaemia. However although her prolactin levels were high 6 months before the exam it normalized 3 months prior to the exam. Hence if hyperprolactinamia was due to exam stress prolactin value should be higher closer to exam instead of becoming normal. Furthermore she was evaluated by the psychiatric team and any co-morbid psychiatric disorder or psychological stress was excluded.

Our patient did not have any menstrual irregularity. In hyperprolactinaemia, amenorrhoea occurs due to inhibition of pulsatile GnRH secretion by increased prolactin levels. For this to happen hyperprolactinaemia should be persistent. But even in persistent hyperprolactinaemia, amenorrhea occurs only in less than 50% [17].

Major drawbacks of our study were not being able to measure multiple prolactin levels with and without stimulatory cues and not performing MRI scan of pituitary during the period of galactorrhoea. This was not possible because patient presented to us 2 weeks after newborn shifted to another location. Unavailability of macroprolactin levels was another drawback. But according to Endocrine Society clinical practice guidelines majority of patients with macroprolactinaemia are asymptomatic and only 20% present with galactorrhoea [16]. Furthermore in our patient prolactin level became normal after 3 months. If the initial elevated level of prolactin was due to macroprolactin prolactin level should have remained high.

To the best of our knowledge, this is the first documented case report of this interesting phenomenon of emotionally induced pseudo-lactation in humans. Transient elevation of serum prolactin during the symptomatic period and its rapid normalization with resolution of symptoms favours the diagnosis of a transient hyperprolactinaemia as the cause for her galactorrhoea. Strong temporal relationship of her symptoms with the presence of newborn in the neighborhood, and, exclusion of other causes on clinical, biochemical and radiological evidence, raise the possibility that transient hyperprolactinaemia was secondary to a transient lactotroph hyperplasia and hyper function which had been triggered by the stimulatory cues from and emotional attachment towards the newborn.

## Conclusion

Though a rare incident, we believe emotionally induced pseudo lactation may be an important secondary cause for transient hyperprolactinaemia in a non-lactating female. Consideration of this as a cause of galactorrhoea may lead to find similar cases in the future. This case illustrates the importance of a thorough clinical history in arriving at a diagnosis.

## Consent

“Written informed consent was obtained from the patient for publication of this Case report and any accompanying images. A copy of the written consent is available for review by the Editor of this journal.”
